# Face and content validity of the virtual reality simulator ‘ScanTrainer®’

**DOI:** 10.1186/s10397-017-1020-6

**Published:** 2017-09-12

**Authors:** Amal Alsalamah, Rudi Campo, Vasilios Tanos, Gregoris Grimbizis, Yves Van Belle, Kerenza Hood, Neil Pugh, Nazar Amso

**Affiliations:** 1 School of Medicine, College of Biomedical and Life Sciences, Cardiff University, Office 220, 45 Salisbury road, Cathays, Cardiff, CF24 4AB UK; 2 European Academy of Gynaecological Surgery, Leuven, Belgium; 3 Aretaeion Medical Center, Nicosia, Cyprus; 4 First Department Obstetrics/Gynecology, Aristotle University of Thessaloniki, Thessaloniki, Greece; 5 Centre for Trials Research, College of Biomedical & Life Sciences, Cardiff University, Cardiff, UK; 6 Department of Medical Physics and Radiology, University Hospital of Wales, Cardiff and Vale University Health Board, Cardiff, UK

**Keywords:** Ultrasound, Validation, Virtual reality simulation, Medical education, Transvaginal ultrasonography, ScanTrainer

## Abstract

**Background:**

Ultrasonography is a first-line imaging in the investigation of women’s irregular bleeding and other gynaecological pathologies, e.g. ovarian cysts and early pregnancy problems. However, teaching ultrasound, especially transvaginal scanning, remains a challenge for health professionals. New technology such as simulation may potentially facilitate and expedite the process of learning ultrasound. Simulation may prove to be realistic, very close to real patient scanning experience for the sonographer and objectively able to assist the development of basic skills such as image manipulation, hand-eye coordination and examination technique.

**Objective:**

The aim of this study was to determine the face and content validity of a virtual reality simulator (ScanTrainer®, MedaPhor plc, Cardiff, Wales, UK) as reflective of real transvaginal ultrasound (TVUS) scanning.

**Method:**

A questionnaire with 14 simulator-related statements was distributed to a number of participants with differing levels of sonography experience in order to determine the level of agreement between the use of the simulator in training and real practice.

**Results:**

There were 36 participants: novices (*n* = 25) and experts (*n* = 11) who rated the simulator. Median scores of face validity statements between experts and non-experts using a 10-point visual analogue scale (VAS) ratings ranged between 7.5 and 9.0 (*p* > 0.05) indicated a high level of agreement. Experts’ median scores of content validity statements ranged from 8.4 to 9.0.

**Conclusions:**

The findings confirm that the simulator has the feel and look of real-time scanning with high face validity. Similarly, its tutorial structures and learning steps confirm the content validity.

**Electronic supplementary material:**

The online version of this article (10.1186/s10397-017-1020-6) contains supplementary material, which is available to authorized users.

## Background

Simulation tools are either simplistic models or complex applications, and regardless of the technology used, a simulator must demonstrate validity to be an effective education tool [1]. This entails gathering evidence from multiple sources to show that the interpretation of image, examination or assessment is sound and sensible [1, 2]. At the outset, validation will usually attempt to confirm the fundamental reasons that these tools need to exist for learning [3–6]. From an educational perspective, a simulated performance should appear realistic when creating a cognitive-sensory mechanism known as ‘sense of presence’ because it allows the trainee/operator to interact with the remote environment as if s/he were present within the environment [7]. With regard to the role of simulation in developing ultrasound knowledge and skills, the validity and reliability of a simulator system for educational goals must be proven, through structured face, content and construct validity studies [1, 8–10].

Face validity is defined as the extent of a simulator’s realism and appropriateness when compared to the actual task [11–13], whereas content validity is defined as the extent to which a simulator’s content is representative of the knowledge or skills that have to be learnt in the real environment. This is based on detailed examination of the learning resources, tutorials and tasks [3, 14–16]. Hence, in the context of ultrasound, face validity addresses the question of how realistic is the simulator, for example, in examining the female pelvis and how realistic is the simulated feel (haptic sensation) experienced during the examination. Similarly, content validity addresses the question of how useful is the ultrasound simulator in learning relevant skills such as measuring endometrial thickness and foetal biometry [13, 17, 18].

According to McDougall and colleagues [4], Kenney and colleagues [19] and Xiao and colleagues [16], face validity is expressed as the assessment of virtual realism by novices, while content validity refers to experts’ assessment of the suitability of a simulator as a teaching tool. However, reports in the literature are diverse and some authors undertake face validity of a simulator by seeking the opinion of any user including expert and non-expert subjects [12, 13, 15, 20–22]. Others have argued that subjects’ experience is required for face validity of any educational instrument [18, 23–26]. With regard to content validity, it widely refers to experts’ judgement towards the learning content and tasks of a simulator [14, 17, 27–29]. Nevertheless, many published studies rely on subjects with different levels of experience in evaluating content validity of a simulator [12, 13, 22, 30–32].

The ultrasound simulator [33] enables the student to acquire transabdominal (TAS) or transvaginal ultrasound scanning (TVUS) skills through a series of simulation tutorials, each with one or more assignments that include specified tasks reflecting real ultrasound practice. Upon completion of the tasks, the simulator provides computer-generated individualised student/trainee feedback. The hypotheses were that the simulator was (1) realistic for the purpose of developing ultrasound skills and reflects real-life scanning and (2) the content of its structured learning approach represents the knowledge and psychomotor skills that must be learnt when scanning patients.

The aim of this study was to determine face and content validity of TVUS ScanTrainer. The objectives were (1) to recruit practitioners with varying levels of ultrasound experience from attendees of an international conference and (2) instruct study volunteers to undertake relevant simulator tutorials and complete a structured questionnaire including statements on face and content validity.

## Methods

**Fig. 1:**
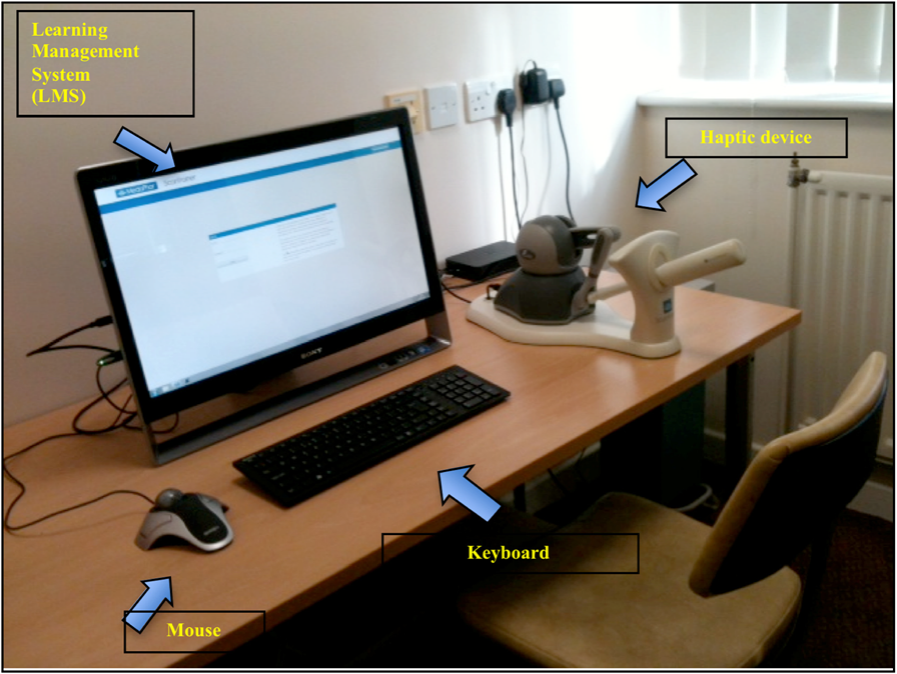
Ultrasound simulator ScanTrainer consists of (1) a monitor which represents learning contents as programmed by specific learning software, and the monitor connects to (2) a haptic device, (3) mouse and (4) keyboard

Subjects were voluntarily recruited from delegates visiting the ‘ESGE Simulation Island’ during the 23rd European Congress of Obstetrics and Gynaecology (2014) in Glasgow, Scotland, UK. Each delegate was given a brief, general introduction on the purpose of the study and instructions on how to use the simulator and the relevant tutorials. They gave verbal consent to participate and proceeded to explore specific tasks in three tutorials with the TVUS ScanTrainer (Fig. 1). These were (1) core skills gynaecology which has assignments on assessing the uterus, ovaries and adnexa and measuring the endometrial thickness, (2) core skills early pregnancy which has assignments on assessing the gestational sac, yolk sac as well as evaluating foetal viability and measurements and (3) advanced skills that consisted of several case studies, e.g. ovarian cyst, ectopic pregnancy and twin pregnancy. At the conclusion of the session, subjects completed a short questionnaire. Participants took between 10 and 15 min to complete the three tutorials.

The structured questionnaire (Additional file 1) consisted of two sections: one detailed subjects’ demographic information, previous ultrasound experience and any previous experience with VR simulation or ultrasound mannequins. The other section included simulation-related statements. An expert was defined as a subject who had ultrasonography experience of nearly 2 years or more, conducted daily scanning sessions and considered her/himself as an independent practitioner. Some experts with many years of independent ultrasound experience had less than daily or weekly sessions due to other commitments. A non-expert was defined as having limited experience with ultrasound, had less than 2 years TVUS experience, with occasional or very limited scanning sessions, e.g. once/month, or considered her/himself as a trainee under supervision, newly qualified or not yet competent in TVUS scanning.

Fourteen simulation-related statements/parameters were subjectively scored along a 10-cm visual analogue scale (VAS) line by marking the point that subjects felt most appropriate, with (0) at one end (very bad) and (10) at the other (very good). Statements 1 to 6 assessed face validity, 7 to 12 evaluated the simulator’s learning content and 13 and 14 were general statements on the value of the simulator as training tool (for practical skill acquisition purpose) and testing tool (for assessment purpose). Ratings on the scale were defined in ‘mm’ as 0–9 (very strongly disagree), 10–19 (strongly disagree), 20–29 (disagree), 30–39 (moderately disagree), 40–49 (mildly disagree), 50 (undecided), 51–59 (mildly agree), 60–69 (moderately agree), 70–79 (agree), 80–89 (strongly agree), 90–100 (very strongly agree). Millimetres were considered for accurate readings of subjects’ marking on scale and later converted to centimetres for final analysis.

The study was conducted in accordance with the general terms and conditions of the South East Wales Research Ethics Committee SEWREC (NHS REC Reference 10/WSE02/75) approval and approval of the study protocol by the congress organising committee.

### Statistical data analysis

IBM SPSS Statistics software version 20.0 was used for statistical analysis. Median values were chosen in preference to mean values as the data were not normally distributed. Median scores and box plots were constructed for each statement as rated by non-experts and experts. Box plots and whiskers represented the median, first and third quartiles, minimum, maximum and outliers of scores obtained by expert and non-expert ratings of the 13 statements. Face validity and general statement items were stratified by expert and non-expert status, while content validity data were reported for experts only. Differences between experts and non-expert ratings were analysed using the Mann-Whitney *U* test using a *p* value ≤ 0.05 to indicate significance.

## Results

Demographic: Thirty-six subjects, 24 females (67%) and 12 males (33%), participated in this pilot study. Nine were UK-based and 27 were based in other European countries. Eleven subjects (31% expert group) rated themselves as skilled with more than 2 years of experience and practiced independently (*n* = 10) or with 1 to 2 years of experience and had daily ultrasound sessions (*n* = 1). Twenty-five subjects (69% non-expert group) were trainees under supervision and included two subjects with more than 2 years TAS experience and limited TVUS scanning. Median age for the expert group was 51 years (range 32–67) and 31 years (range 25–39) for the non-expert group. The median ultrasound experience for experts was more than 2 years and for non-experts was 6 to 11 months. Further breakdown of demographics and years of ultrasound experience is detailed in Table 1.

**Table 1:** Participants’ demographics and ultrasonography experience

	Non-expert	Expert
No. of participants (*n* = 36)	25 (69%)	11 (31%)
Gender		
Female	17 (68%)	7 (64%)
Male	8 (32%)	4 (36%)
Country of practice		
Within UK	6 (24%)	3 (27%)
Outside UK	19 (75%)	8 (73%)
Speciality		
Consultant	–	3 (27%)
Obs/Gyn specialist	2 (8%)	4 (36%)
Specialist trainee	20 (80%)	3 (27%)
Medical student	1 (4%)	–
Radiographer	–	1 (10%)
Other (midwives)	2 (8%)	–
Median age	31 (25–39)	51 (32–67)
Years of ultrasound experience		
Never	3 (12%)	–
< 6 months	5 (20%)	–
6–11 months	9 (36%)	–
1–2 years	6 (24%)	1 (10%)
> 2 years	2 (8%)	10 (90%)
Transvaginal ultrasound experience		
Independent practitioner	2 (8%)	11 (100%)
Trainee under supervision	23 (92%)	–
Ultrasound sessions		
Never	4 (16%)	–
Daily	1 (4%)	5 (46%)
Once/week	9 (36%)	–
Once/month	3 (12%)	2 (18%)
Occasionally	5 (20%)	2 (18%)
Other	3 (12%)	2 (18%)
Previous experience with the ScanTrainer®		
Yes	3 (12%)	3 (27%)
No	22 (88%)	8 (73%)
Previous experience with ultrasound model, i.e. blue Phantom™		
Yes	4 (16%)	4 (36%)
No	21 (84%)	7 (64%)

**Fig. 2:**
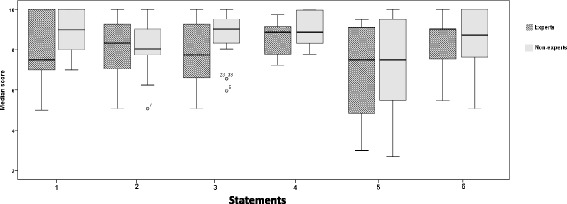
Box plots represented the median, first and third quartiles, minimum, maximum and outliers of scores obtained by expert and non-expert ratings of the six face validity statements. Dots (outliers) represented those experts who scored lower than others and the number referred to participant’s code number in data analysis and that did not relate to score value

**Fig. 3:**
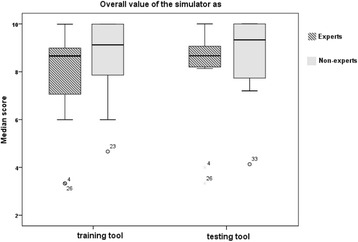
Box plots represented the median, first and third quartiles, minimum, maximum and outliers of scores obtained by expert and non-expert ratings of the two general validity statements on the simulator as training and testing tool. Dots (outliers) represented those experts who scored lower than others and the number referred to participant’s code number in data analysis and that did not relate to score value

Face validity: Median scores of face validity statements are detailed in Table 2. In summary, experts’ and non-experts’ ratings ranged between 7.5 and 9.0 and were slightly higher than those by experts in two statements (2 and 6) relating to ‘realism of the simulator to simulate the TVUS scan of female pelvis and realism of the simulator to provide actual action of all buttons provided in the control panel’. Two statements (1 and 3) were rated lower by experts and related to ‘relevance of the simulator for actual TVUS scanning and the realism of the simulator to simulate the movements possibly required to perform in the female pelvic anatomy (uterus, ovaries/adnexa, Pouch of Douglas POD)’. The remaining two statements (4 and 5) referring to ‘realism of the ultrasound image generated during the performance and force feedback provided on the operator’s hand to simulate real scan’ were equally rated. Two general statements (13 and 14) were also rated lower by experts. However, there were no statistically significant differences between the two groups’ ratings in all statements (Table 1). Median values and box plots of the eight statements in the two groups are shown in Figs. 2 and 3.

**Table 2:** Face validity ‘median scores’ ratings by experts and non-experts (*n* = 36)

	Median score (range)			
Face validity statements	Expert (*n* = 11)	Non-expert (*n* = 25)	Overall	*p* value
Statement 1: Relevance of the simulator for actual transvaginal ultrasound scanning	7.5 (5.0–10)	9.0 (7.0–10)	8.7 (5.0–10)	0.1
Statement 2: Realism of the simulator to simulate the transvaginal scan of female pelvis	8.3 (5.0–10)	8.0 (5.9–10)	8.1 (5.0–10)	0.9
Statement 3: Realism of the simulator to simulate the movements possibly required to perform in the female pelvic anatomy (uterus, ovaries/adnexa, POD)	7.7 (1.0–10)	9.0 (5.0–10)	9.0 (1.0–10)	0.1
Statement 4: Realism of the ultrasound image generated during the performance	9.0 (1.3–9.8)	9.0 (6.0–10)	9.0 (1.3–10)	0.2
Statement 5: Force feedback provided on the operator’s hand to simulate real scan	7.5 (3.0–9.5)	7.5 (2.7–10)	7.5 (2.7–10)	0.4
Statement 6: Realism of simulator to provide actual action of all buttons provided in the control panel	9.0 (1.0–10)	8.7 (3.0–10)	9.0 (1.0–10)	0.5
General statements				
Statement 13: Overall value of the simulator as a training tool	9.0 (5.0–10)	9.3 (6.0–10)	9.0 (5.0–10)	0.2
Statement 14: Overall value of the simulator as a testing tool	9.0 (5.0–10)	9.5 (5.6–10)	9.3 (5.0–10)	0.2

**Fig. 4:**
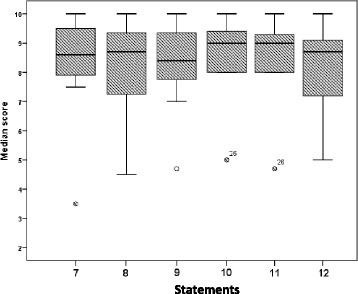
Box plots represented the median, first and third quartiles, minimum, maximum and outliers of scores obtained by expert and non-expert ratings of the six content validity statements. Dots (outliers) represented those experts who scored lower than others and the number referred to participant’s code number in data analysis and that did not relate to score value

Content validity: Experts’ median scores of content validity statements ranged from 8.4 to 9.0 and are detailed in Table 3. Median values and box plots of the six statements are shown in Fig. 4.

**Table 3:** Content validity ‘median scores’ ratings by experts (*n* = 11)

Content validity statements	Expert median (range)
Statement 7: Realism of the simulator to provide the endometrial thickness measurement in gynaecology task	8.6 (3.5–10)
Statement 8: Realism of the simulator to provide measurements of the ovary in gynaecology task	8.7 (4.5–10)
Statement 9: Ability to test normal gynaecological anatomy: uterus, adnexa and Pouch of Douglas	8.4 (4.7–10)
Statement 10: Ability to test early pregnancy structures: fetus, viability and placenta	9.0 (5.0–10)
Statement 11: Realism of the simulator to provide the CRL measurement in early pregnancy task	9.0 (4.7–10)
Statement 12: Relevance of the simulator’s learning resource, videos and ScanTutor function	8.7 (5.0–10)

## Discussion

In this study, the ScanTrainer® simulator demonstrated high face and content validity and its overall value as a training and testing tool received high ratings as well. To accurately measure participants’ level of agreement with relevant statements, VAS method was used in the questionnaire [34]. Higher ratings were given by non-experts than experts with regard to ‘relevance of the simulator to actual TVUS’ and *‘*its realism to simulate the movements required to perform in the examination of the female pelvis’ (statements 1 and 3) highlighting the fact that such realism is crucial for non-experts for several reasons. This may be because experts need to develop greater understanding of the strengths and limitations of the simulator compared to trainees [35]. Alternatively, beginners in the early stages of learning ultrasound skills are able to address their learning needs through simulated learning compared to the experts who expect variety and advanced or more complex performance rather than basic tutorials [12].

There are no comparable ‘face and content’ validity studies addressing virtual reality simulators for TVUS in obstetrics and gynaecology have been published in the literature. In a face validity study of the dVT robotic surgery simulator, experts rated the simulator as less useful for training experts than for students/juniors and pointed out to the experts’ need for more critical and advanced procedures in gynaecological surgery and that simulators specifically designed for learning basic skills are less preferable to experts [32]. Creating simulated scenarios to correspond to real ones is always a challenge [3, 29, 36, 37].

Experts’ ratings were higher for two statements relating to the realism of the simulator to simulate the TVUS scan of a female pelvis and in providing actual action of all buttons in the control panel (statements 2 and 6) This may stem from non-experts’ limited knowledge and experience, or they might not be familiar with the measurement possibilities of virtual simulators [20, 23]. Similarly, Weidenbach and colleagues [1] argued that experts gave a better grading for the realism of the EchoCom echocardiography simulator because they were not distracted to drawbacks such as mannequin size and its surface properties, which were harder and more slippery than the human skin, and that experts scanned more instinctively. The author noted that this mental flexibility seemed to be as yet underdeveloped in beginners.

Non-experts’ and experts’ ratings were similar when evaluating the realism of the ultrasound image generated during the performance and the force feedback provided onto the operator’s hand (statements 4 and 5). Force feedback (haptics) scored 7.5 out of 10, the lowest score in this study. Similar to this study, Chalasani and colleagues [38] reported low face validity ratings for the haptic force-feedback device of a transrectal ultrasound TRUS-guided prostatic biopsy virtual reality simulator (experts’ lifelike rating 64% and novices’ 67%) even though the author pointed out that haptics, often very difficult to replicate in a simulator environment, were realistic. Haptics will not replace the real-patient scan experience but should enhance the learning approach and improve self-confidence. A further factor is that the ScanTrainer’s haptic device can be tailored to three force feedback levels: normal resistance (most realistic), reduced and minimal (lowest) designed to avoid overheating during heavy use, and it is likely that a lower force feedback setting might have contributed to the lower scores.

The role of force feedback in laparoscopic surgery is not clear [20]. Improving the realism of the simulator and its anatomical structures increases costs considerably due to increased demands for more complex hardware and software. In contrast, Lin and colleagues [39] encouraged learning of bone-sawing skills with simulators that provide force feedback rather than not, confirming the importance of force feedback when seeking to enhance hand-eye coordination. With regard to ScanTrainer, virtual ultrasound and haptics are used instead of a mannequin allowing measurement of the force applied to the probe and provide a somewhat realistic force-feedback during scanning. However, it still has the limitation of allowing a lower range of movements to the probe while lacking a simulated environment exemplified by the absence of a physical mannequin [40].

There are numerous simulator systems in usage particularly in the fields of laparoscopy and endoscopy, and several authors emphasised the importance of evaluating their content, including reviewing each learning task and assessing its overall value to determine whether it is appropriate for the test and whether the test contains several steps and skills for practice [12, 17, 31, 38]. In this study, experts’ data were used to assess content validity. They had adequate time to review the simulator’s learning resources, help functionality ‘ScanTutor’, read the task-specific instructions and undertake specified tasks before going on to the next step in the same tutorial. In addition, participants had the opportunity to review feedback on their performance in the respective tasks. The results of this study demonstrated that the simulator’s content and metrics were appropriate and relevant for ultrasound practice.

There are a number of published content validity studies in ultrasound simulation, such as the educational curriculum for ultrasonic propulsion to treat urinary tract calculi [41], web-based assessment of the extended focused assessment sonography in trauma (EFAST) [2] and validation of the objective structured assessment of technical skills for duplex assessment of arterial stenosis (DUOSATS) [42] which is not based on virtual reality simulator devices. Shumard and colleagues [43] reported on face and content validity of a novel second trimester uterine evacuation task trainer designed to train doctors to perform simulated dilatation and evacuation under ultrasound guidance. Although all respondents were residents with limited ultrasound experience, they rated the task trainer as excellent.

Other studies evaluated the effectiveness of simulation-based training in obstetrics and gynaecology ultrasound, whether to investigate the construct validity of a simulator system [9, 40, 44, 45] or to compare simulation training to conventional methods such as theoretical lectures and hands-on training on patients [10, 46].

Feedback that is automatically generated immediately after a practical simulator session should enhance trainees’ knowledge and ability to reflect critically on their performance and improve their skills [47]. However, the big challenge is to determine how accurate, realistic and trusted the feedback is and, thus, should also be validated appropriately.

Validation studies at national scientific meetings have been reported previously [25, 48]. They offer researchers a rich environment where subjects from different backgrounds and levels of experience are present in one place at the same time. A potential limitation of the study is that it did not determine in advance the sample size required to obtain a reliable result for face and content validation. There is no agreement on the adequacy of sample size in such studies [12, 13]. The number of subjects in this study was higher, and the findings are consistent with others [18, 22, 31, 49]. In addition, many face and content validity studies of simulators were based on smaller sample size compared to the current study [13, 19, 30, 36, 50, 51]. A larger number of participants in this study might have improved the confidence in the results [2]. Participants in this study were from different UK and European institutions unlike others who were from single academic institution [41]; thus, it may be more widely generalizable.

## Conclusions

In summary, this study confirms that ScanTrainer simulator has the feel and look (face validity) and tutorial structure (content validity) to be realistic and relevant for actual TVUS scanning. This study also concurs with the notion that advancing computer technologies have been able to incorporate virtual reality into training to facilitate the practice of basic skills as well as complex procedures that leave little room for error or mistake [3, 10, 24, 20]. Equally, such simulators should be part of the skill training labs in teaching hospitals as it is recommended for endoscopic surgery [52, 53]. It should be subject to an ongoing validation to address trainees’ learning needs, provide a structured training path and provide validated test procedures with the global and final aim to improve patient care and safety [30, 31, 36, 52].

## Additional file

Additional file 1: Face Validity Questionnaire: the ScanTrainer Ultrasound Simulator. (DOCX 207 kb)
