# Supplementary material for: Face and content validity of the virtual reality simulator ‘ScanTrainer®’
Source: Gynecol Surg. 2017 Sep 12;14(1):18. doi: 10.1186/s10397-017-1020-6 (PMC5596038; doi:10.1186/s10397-017-1020-6)
Supplement: Additional file 1: — Face Validity Questionnaire: the ScanTrainer Ultrasound Simulator. (DOCX 207 kb) [file 10397_2017_1020_MOESM1_ESM.docx]

Please use Visual Analogue Score (VAS) to rate all questions
by placing an “X” where appropriate e.g.

0 (very bad) X 10 (very good)

| 1. Relevance of the simulator for actual transvaginal ultrasound scanning |
| --- |
| 0 10 |
| 1. Realism of the simulator to simulate the transvaginal scan of female pelvis |
| 0 10 |
| 1. Realism of the simulator to simulate the movements possibly required to perform in the female pelvic anatomy (uterus, ovaries/adnexa, POD) |
| 0 10 |
| 1. Realism of the ultrasound image generated during the performance |
| 0 10 |
| 1. Force feedback provided on the operator’s hand to simulate real scan |
| 0 10 |
| 1. Realism of simulator to provide actual action of all buttons provided in the control panel |
| 0 10 |
| 1. Realism of the simulator to provide the endometrial thickness measurement in gynaecology task |
| 0 10 |
| 1. Realism of the simulator to provide measurements of the ovary in gynaecology task |
| 0 10 |
| 1. Ability to test normal gynaecological anatomy: uterus, adnexa and Pouch of Douglas |
| 0 10 |
| 1. Ability to test early pregnancy structures: Fetus, viability and placenta |
| 0 10 |
| 1. Realism of the simulator to provide the CRL measurement in early pregnancy task |
| 0 10 |
| 1. Relevance of the simulator’s learning resource, videos and ScanTutor function |
| 0 10 |
| 1. Overall value of the simulator as a training tool |
| 0 10 |
| 1. Overall value of the simulator as a testing tool |
| 0 10 |

Details about yourself **(tick relevant box or provide written answers):**

**Name:**

**Age: Gender:** Female Male

**Hospital/ Department:**

1. **State the grade/band /post you currently hold?**

Consultant _________________ Specialist trainee _________________

Specialist _________________ years of training ST_________________

Nurse Hysteroscopist Medical student _________________

Other (please specify) _________________

Country of practice _________________

1. **State the number of years you have been practising ultrasound in clinics:**

Never < 6 months 6 – 11 months 1-2 years > 2 years

1. **Clarify your transvaginal ultrasonography experience, you are….**

Independent practitioner Trainee under supervision Trainer/tutor

Other (*please describe)* _________________

1. **How often do you scan?**

Never Daily Once a week Once a month Occasionally

Other (*please describe)* _________________

1. **Previous experience with ScanTrainer ultrasound simulator?** Yes No
2. **Previous experience with any other ultrasound models?** Yes No
